# The Wasserman Reaction in Syphilitic Eye Lesions

**Published:** 1910-03

**Authors:** 


### The Wasserman Reaction in Syphilitic Eye Lesions.

The Chicago Ophthalmological Society recently held a symposium on syphilis, at which Dr. Harris explains in detail the Wasserman test and drew the following conclusions:

1. That spirochæta pallida are present in all syphilitic lesions, including those of the eye.
2. In chancres and mucous patches the diagnosis should be made by the demonstration of the spirachæta.
3. All other lesions of the eye of syphilitic origin may be diagnosed by means of the Wasserman test.
4. Eye conditions depending upon pathological changes in the nervous system of syphilitic origin may be diagnosed by the Wasserman test.
5. All doubtful cases that might be explained on a syphilitic basis should be given the Wasserman test.—*Med. Review of Reviews.*

---

The present reckoning of the season's football disasters shows 30 deaths, 216 players injured, 12 broken collar bones, 8 broken noses, 12 broken legs, 19 broken ankles, 13 broken shoulders, 8 broken wrists, 8 broken fingers, 6 broken hands and 3 broken jaws. This is not the summary of the casualties of a battle. It is the score of an athletic game, styled a sport, a stimulus to manhood, a developer of youthful brawn and a builder of character. It is the price that has been paid by the boys of the United States for a little glory, much bitter disappointment and a measure of demoralizing public entertainment.—*Exchange.*

